# Vertical transmission of *Bartonella schoenbuchensis* in *Lipoptena cervi*

**DOI:** 10.1186/s13071-015-0764-y

**Published:** 2015-03-21

**Authors:** Arnout de Bruin, Arieke Docters van Leeuwen, Setareh Jahfari, Willem Takken, Mihály Földvári, László Dremmel, Hein Sprong, Gábor Földvári

**Affiliations:** Centre for Zoonoses & Environmental Microbiology, Centre for Infectious Disease Control, National Institute for Public Health and the Environment, Bilthoven, the Netherlands; Laboratory of Entomology, Wageningen University, Wageningen, the Netherlands; MTA-DE ‘Lendulet’ Behavioural Ecology Research Group, Department of Evolutionary Zoology, University of Debrecen, Debrecen, Hungary; Institute of Wildlife Management and Vertebrate Zoology, Faculty of Forestry, University of West Hungary, Sopron, Hungary; Department of Parasitology and Zoology, Faculty of Veterinary Science, SzentIstván University, Budapest, Hungary

**Keywords:** *Lipoptena cervi*, Deer ked, Pathogen, Vector, *Anaplasma*, *Bartonella*, *Rickettsia*, *Ixodes ricinus*

## Abstract

**Background:**

*Lipoptena cervi* (Diptera: Hippoboscidae) is a hematophagous ectoparasite of cervids, which is considered to transmit pathogens between animals and occasionally to humans. The principal life stage that is able to parasitize new hosts is a winged ked that just emerged from a pupa. To facilitate efficient transmission of pathogens between hosts, vertical transmission from female deer keds to their offspring is necessary. We investigated vertical transmission of several vector-borne pathogens associated with cervids.

**Methods:**

Deer keds from several locations in Hungary were collected between 2009 and 2012. All life stages were represented: winged free-ranging adults, wingless adults collected from *Capreolus capreolus* and *Cervus elaphus,* developing larvae dissected from gravid females, and fully developed pupae. The presence of zoonotic pathogens was determined using qPCR or conventional PCR assays performed on DNA lysates. From the PCR-positive lysates, a gene fragment was amplified and sequenced for confirmation of pathogen presence, and/or pathogen species identification.

**Results:**

DNA of *Bartonella schoenbuchensis* was found in wingless males (2%) and females (2%) obtained from *Cervus elaphus*, dissected developing larvae (71%), and free-ranging winged males (2%) and females (11%). DNA of *Anaplasma phagocytophilum* and *Rickettsia* species was present in *L. cervi* adults, but not in immature stages. DNA of *Candidatus* Neoehrlichia mikurensis was absent in any of the life stages of *L. cervi*.

**Conclusions:**

*B. schoenbuchensis* is transmitted from wingless adult females to developing larvae, making it very likely that *L. cervi* is a vector for *B. schoenbuchensis. Lipoptena cervi* is probably not a vector for *A. phagocytophilum*, *Rickettsia* species, and *Candidatus* N. mikurensis*.*

## Background

*Lipoptena cervi* (deer ked) is an obligate hematophagous ectoparasite of cervids and domesticated animals [1,2], which occasionally bites humans [3]. This species has a Palearctic distribution and belongs to a highly specialized family of flies (Diptera: Brachycera), called louse flies (Hippoboscidae) [4,5]. Within the family Hippoboscidae, a few species are known to transmit zoonotic pathogens or protozoan parasites to wildlife, or domesticated animals [6-8].

In general, *L. cervi* is considered a mere nuisance for animals only [9], and clinical symptoms after they bite humans are not very severe [10,11]. However, *L. cervi* is also considered a potential vector for a number of zoonotic pathogens, such as *Bartonella*, *Anaplasma*, and *Rickettsia* species [12-14]. *Lipoptena cervi* shows a number of interesting life history traits, in which vertical transmission of pathogens from females to offspring seems to be essential to facilitate efficient transmission between vertebrate hosts. The life cycle of *L. cervi* starts (arbitrarily) with free-ranging winged adult deer keds that search for suitable (cervid) hosts. After landing on a host, they crawl into the fur, shed their wings and thereby become permanently associated with that host. The wingless deer keds frequently take blood meals from that host, which is soon followed by mating. After that, a larva will develop (one at a time) that is retained by the female until the third instar. This third instar larva is deposited on the cervid fur as a white prepupa, which immediately starts to pupate. The fully developed and darkened pupa drops to the ground and remains there until August-September, after which a new generation of winged adult keds can emerge [2,15].

When in search for a host, winged adult *L. cervi* are attracted to large moving dark colored objects [3], fly short distances only, and often attack unsuitable hosts (e.g. humans) [15]. The loss of its wings, invoked by crawling through the fur of a host [15], makes any subsequent host switch of *L. cervi* difficult or impossible [16]. However, it was suggested that wingless adults from another hippoboscid species, Neotropical deer ked *L. mazamae,* can be transmitted mechanically from female white-tailed deer to their offspring [17]. Nevertheless, vertical transmission of pathogens from female deer keds to their offspring is most likely a prerequisite for efficient vector potential.

A number of studies show that various zoonotic pathogens are present in a number of the mentioned life stages of *L. cervi. Bartonella schoenbuchensis,* causing deer ked dermatitis in humans, was isolated from the gut of wingless adult *L. cervi* [10]. Although detection of *B. schoenbuchensis* in wingless adult deer keds was confirmed by others as well [13,18], DNA of this particular pathogen has not been detected in immature *L. cervi* life stages. However, DNA of *Bartonella* species in general, including potential endosymbionts, was detected in *L. cervi* wingless adults [12,18], fully developed pupae [8,12], and free-ranging winged deer ked adults [8]. These findings at least support vector potential of *L. cervi* for (zoonotic) *Bartonella* species.

In addition to *Bartonella*, *Anaplasma* species may be transmitted by *L. cervi* as well. *Anaplasma ovis,* which causes anaplasmosis primarily in sheep, was detected in a single free-ranging winged adult deer ked. However, none of the additional adult wingless deer keds collected from roe and red deer tested positive for *Anaplasma* spp. [14]. In contrast to *A. ovis*, *A. phagocytophilum* can cause anaplasmosis both in sheep and cattle, and a zoonotic disease referred to as granulocytic anaplasmosis in humans [19,20]. This pathogen was detected in *L. cervi* wingless adults, collected from cervids that tested negative for this pathogen, but not in free-ranging winged adult deer keds [21]. In another study, wingless adult deer keds, obtained from culled roe deer, were found positive for *A. phagocytophilum* as well [22]. Additional molecular typing revealed that *A. phagocytophilum* ecotype II was found especially in roe deer, *Ixodes ricinus* ticks, and deer keds.

Finally, vector potential of *L. cervi* for *Rickettsia* species was studied as well. Rickettsiae are non-motile, Gram-negative, highly pleomorphic bacteria, which can be transmitted by competent arthropod vectors, such as ticks, lice, and fleas, and can cause a large number of zoonotic diseases in humans [23]. *Rickettsia helvetica* and other unidentified *Rickettsia* species were detected in pools of wingless adult *L. cervi,* collected from roe deer and red deer, but not in winged free-ranging adult deer keds [14]. Although pathogenic potential of *R. helvetica* is still unclear, this pathogen has been associated with acute perimyocarditis [24], an unexplained febrile illness [25], and recently also with meningitis [26].

In this study, the vector potential of *L. cervi* was further explored for four zoonotic pathogens: *Candidatus* Neoehrlichia mikurensis*, A. phagocytophilum*, *Bartonella* spp., and *Rickettsia* spp. We tested winged free-ranging adults, wingless adults collected from roe deer and red deer, developing larvae dissected from females, and pupae collected from the fur of cervids for the presence of these zoonotic pathogens.

## Methods

### Collection of *Lipoptena cervi* and DNA extraction procedures

Between 2009 and 2012, 345 deer keds and fully developed pupae were collected from different locations in Hungary. A total of 248 wingless adult deer keds (males and females), and three developed pupae were collected from one male and ten female *Cervus elaphus* hosts, and two *Capreolus capreolus* hosts (one male and one female). In addition, 94 free-ranging winged adult deer keds (52 males and 42 females) were collected during the same time period from various locations in Hungary. No ethical approval is required for the experimental methods used in this study.

In the laboratory, seven wingless adult female deer keds were dissected, and developing larvae were harvested. For all samples, DNA was extracted first by alkaline lysis as described earlier [27]. For a number of samples that showed coloration or a turbid DNA suspension after alkaline lysis, a Qiagen DNA extraction procedure [28] was performed to further purify DNA from co-extracted substances that may inhibit downstream (q)PCR reactions.

### PCR assays and sequencing procedures

Extracted DNA was tested for the presence of four zoonotic pathogens: *Candidatus* N. mikurensis*, A. phagocytophilum*, *Bartonella* spp., and *Rickettsia* spp. For detection of *A. phagocytophilum* and *Candidatus* N. mikurensis DNA*,* a single multiplex qPCR assay was used, which targets specific regions of genes *msp2* (Major Surface Protein 2) for *A. phagocytophilum*, and *groEL* (heat shock protein) for *Candidatus* N. mikurensis. This was followed by conventional PCR and sequencing part of the *groEL* gene for samples positive for *A. phagocytophilum.* Detection of *A. phagocytophilum* and *Candidatus* N. mikurensis DNA by qPCR, and confirmation by sequencing of positive samples were performed as described earlier [22,28]. For detection of *Bartonella* spp. a conventional PCR assay was used, which targets a part of the citrate synthase gene (*gltA*). This was followed by sequencing of positive samples for species identification. Both conventional PCR and sequencing procedures were performed, as described earlier [29].

For detection of *Rickettsia* species, we used a multiplex qPCR assay, in which two different regions of the *gltA* gene are targeted. We designed primers and probes to amplify a region of the *gltA* gene, specific for *R. helvetica* (Table 1)*.* This assay was combined with primers and probes designed by Stenos *et al.,* which amplify a different region of the *gltA* gene for the detection of *Rickettsia* species in general [30]. All qPCR runs were carried out in a final volume of 20 μl containing IQ Multiplex Powermix (Bio-Rad), and 400 nM of primers and hydrolysis probes. Conditions for PCR amplification were the following: 95°C for 5 min, 60 thermocycles at 95°C for 5 s, and 60°C for 35 s, followed by a final incubation step at 37°C for 20 s. PCR assays were carried out on a LightCycler 480 instrument (Roche Diagnostics Nederland B.V, Almere, the Netherlands), and analysis was performed on the instrument’s software (release 1.5.1.62). Quantification cycle (Cq) values were calculated using the second derivative method. For samples positive for *Rickettsia* DNA in qPCR, conventional PCR was performed on a *gltA* region, using forward and reverse primers CS490 and Rp1258n as described by Roux *et al.* [31]. PCR amplification was carried out using the HotStarTaq master mix (Qiagen, Westburg, Germany), and 400 nM primers in a total reaction volume of 25 μl. Thermocycling conditions were the following: 95°C for 15 min, 40 cycles at 94°C for 30 s, 54°C for 30 s, and 72°C for 55 s, followed by a final step at 72°C for 7 min. We included three μl of DNA template. Conventional PCRs were carried out in a P × 2 thermal cycler (Thermo Electron Corporation, Breda, the Netherlands). PCR products were visualized on a 2% agarose gel, and sequenced by BaseClear according to the company’s protocol. BLAST analysis was performed to confirm *Rickettsia* species identification.

**Table 1:** **Newly developed primers and probe** ***for Rickettsia helvetica*** **targeting a region of the** ***gltA*** **gene**

**Primers & probe**	**Oligo name**	**Primer and probe sequences (5′- > 3’)**	**Product length**
forward primer	Rick_HelvgltA_F2	ATGATCCGTTTAGGTTAATAGGCTTCGGTC	123 bp
reverse primer	Rick_HelvgltA_R2	TTGTAAGAGCGGATTGTTTTCTAGCTGTC	
Probe (Atto425)	Rick_HelvgltA_pr3	ATTO425-CGATC + C + ACG + TG + CCGCAGT-BHQ1 (+ = LNA)	

LNA = Locked Nucleic Acid, indicated by symbol ‘+’.

Using Fisher’s exact test on 2 × 2 contingency tables, we tested if there was a significant difference between numbers of male and female deer keds positive to *Bartonella* and *A. phagocytophilum.*

## Results and discussion

*Bartonella* DNA was detected in all life stages of *L. cervi*, except the three fully developed pupae (Table 2). We detected *Bartonella* DNA in 182 of ked samples, including wingless adult males and females collected from both red deer and roe deer, free ranging winged males and females, and larvae harvested from adult females. Sequencing part of the *gltA* gene revealed the presence of *Bartonella schoenbuchensis* DNA in free-ranging winged adults (one male and five females), wingless adults (two males and three females), and five harvested developing larvae (Table 2). These findings indicate that *Bartonella* is transmitted vertically from wingless females to larvae that will develop into pupae. However, *Bartonella* DNA was not detected in the three pupae collected from red or roe deer, probably due to the small sample size. Although we were not able to detect *Bartonella* in fully developed pupae, it is very likely that *L. cervi* is a vector for this pathogen. Other studies showed that *Bartonella* DNA was present in various *L. cervi* life stages as well [8,10,12,13,18]. In addition, it was reported that another hippoboscid species (*Melophagus ovinus*) is able to transmit *Bartonella* vertically from females to their offspring [13].

**Table 2:** **Detection of vector-borne pathogens in various life stages of** ***L. cervi***

**Description**	***L. cervi*** **life stage**	**Samples**	***A. phagocytophilum***	***Rickettsia*** **spp.**	***R. helvetica***	***Bartonella*** **spp.**	***B. schoenbuchensis***
Red deer	Wingless male	97	49 (51%)	6 (6%)	3 (3%)	56 (58%)	2 (2%)
	Wingless female	125	70 (56%)	18 (14%)	7 (6%)	95 (76%)	3 (2%)
	Developed pupae	2	-	-	-	-	-
Roe deer	Wingless male	7	-	-	-	2 (29%)	-
	Wingless female	13	-	-	-	2 (15%)	-
	Developed pupae	1	-	-	-	-	-
Free-ranging	Winged male	52	1 (2%)	2 (4%)	-	6 (12%)	1 (2%)
	Winged female	42	1 (2%)	3 (7%)	1 (2%)	9 (21%)	5 (12%)
Dissection	Developing larvae	7	-	-	-	6 (86%)	5 (71%)
	Remnants from females	6	2 (33%)	-	-	6 (100%)	-
Total		352	123	29	11	182	16

*Anaplasma phagocytophilum* DNA was detected in 123 (35%) of deer ked samples, 119 were wingless adults collected from red deer, two were free-ranging winged adults, and two remnants of female adult deer keds after dissection and removal of developing larvae (Table 2). We were able to confirm the presence of *A. phagocytophilum* by conventional PCR and sequencing in five wingless adult females and six wingless adult males. We found no *A. phagocytophilum* DNA in adult deer keds collected from roe deer, pupae collected from both red deer and roe deer, or in larvae harvested from adult females. Since the majority of *A. phagocytophilum*-positive deer ked samples were wingless adults, and we found no *Anaplasma* DNA in developing larvae and fully developed pupae, vertical transmission of this pathogen from females to offspring is not very likely. In addition, the number of positive winged adults is also quite limited and presence of *A. phagocytophilum* DNA could not be confirmed by sequencing. The finding of *Anaplasma*-positive winged *L. cervi* adults in combination with the absence of positive larvae and pupae is rather puzzling. It may be possible that these winged *L. cervi* have taken a blood meal from another (unsuitable) host, retained their wings somehow, and were able to search for another more suitable host. Other studies reported the presence of *A. phagocytophilum* in only wingless adults as well [21,22], and together with our results, this indicates that *L. cervi* is probably not involved in the transmission of *A. phagocytophilum.*

For *Rickettsia* spp., 29 deer ked samples were found positive for a *gltA* sequence detected using primers and probes designed by Stenos *et al.* [30]. In addition, 11 deer ked samples were positive for *Rickettsia helvetica,* using primers and probes designed specifically for a *gltA* region of this species. Four deer ked samples showed positive results based on both *gltA* regions. *Rickettsia* DNA was detected in wingless *L. cervi* males and females, originating from red deer, and in free-ranging winged deer keds. However, qPCR assay results indicated very low levels of *Rickettsia* DNA present within the samples. Therefore, we were not able to confirm *Rickettsia* DNA presence by subsequent conventional PCR and sequencing. As for *A. phagocytophilum*, dissected developing larvae and fully developed pupae showed no positive results for *Rickettsia* DNA, which indicates that vertical transmission of *Rickettsia* species by *L. cervi* females is not very likely. The absence of vertical transmission and the presence of *Rickettsia* species in winged and wingless deer keds only indicate that *L. cervi* is probably not involved in the transmission of rickettsiae*.*

We detected no *Candidatus* N. mikurensis DNA in any of the deer ked life stages collected. According to literature, *Candidatus* N. mikurensis is primarily transmitted by ticks [28,32], and we found no reports in which this pathogen was transmitted by other vectors. In addition, *Candidatus* N. mikurensis can be found in several rodent species, which may act as reservoir hosts [28,33-35]. However, we found no reports of this pathogen present in deer. Red deer and roe deer are incompetent hosts for *Borrelia burgdorferi* s.l. genospecies [36,37], and are even known to be able to reduce *Borrelia* infections in *I. ricinus* ticks [38,39]. Therefore, it may also be possible that the same deer species are incompetent reservoir hosts for *Candidatus* N. mikurensis as well. Together with our findings, this indicates that although *L. cervi* and ticks (*I. ricinus*) can share a common host (cervids), *L. cervi* is probably not involved in the transmission of *Candidatus* N. mikurensis.

On red deer, we observed larger numbers of positive wingless females in comparison to positive wingless males for all three pathogenic genera. For *Bartonella* and *Rickettsia* species, the difference between wingless positive females and males was significant (*p* = 0.005 and *p* = 0.038, respectively). Free-ranging females also had a higher prevalence for these two bacteria, however not significantly due to low sample size. This is an unexpected difference, since it is reported that when unfed, both male and female deer keds have the same weight, and when blood-fed, males are heavier than females [40]. Therefore, the significantly more infected females cannot be the result of a larger blood meal compared to males. One explanation is that *Rickettsia* and *Bartonella* species (zoonotic or endosymbionts) are able to colonize and/or survive in females more efficiently than in males. Since vertical transmission of pathogens in *L. cervi* is necessary for efficient transmission between vertebrate hosts, strong selection toward infection of female deer keds can be expected. However, a (molecular) mechanism for this is still unknown.

Another possibility is the involvement of endosymbionts in this phenomenon. In common with other insects, in which all the life stages are dependent solely on blood as the nutrient source, hippoboscids have symbionts. These symbionts are housed in a mycetome on the intestine and are transferred to offspring accompanied to nutrients provided by the mother for her intra-uterine larva [41]. Many maternally inherited endosymbionts manipulate their host’s reproduction in various ways to enhance their own fitness. One such mechanism is male killing, in which sons of infected mothers are killed by the endosymbiont during development as described for *Wolbachia* in another dipteran, *Drosophila innubila* [42]. Nycterophiliine bat flies (Diptera, Streblidae), belonging to a related parasitic family to louse flies, were recently shown to have *Wolbachia* endosymbionts [43] but, unfortunately, hippoboscid flies were not examined for these, only for other endosymbionts [44]. Either in co-occurrence with endosymbionts or functioning as endosymbionts themselves, *Bartonella* and/or *Rickettsia* spp. infection in female deer ked might lead to more female offspring compared to uninfected females, possibly resulting in the observed asymmetry in the female: male ratio of infected individuals.

Finally, we were not able to test blood or tissue samples of roe or red deer, from which the wingless adult deer keds were collected for presence of pathogens. However, cervids are known reservoir hosts for at least a number of zoonotic pathogens we investigated [45-49].

## Conclusions

Detection of pathogens in wingless females, developing larvae, and fully developed pupae indicates vertical transmission from female *L. cervi* to their offspring. The only deer ked life stage that is able to search actively for new hosts is a winged adult following eclosion from a pupa. Therefore, vertical transmission and detection of pathogens in emerged winged adults is essential to show vector potential of *L. cervi*.

*Anaplasma phagocytophilum*, or *Rickettsia* species were not present in harvested developing larvae or fully developed pupae, and only a limited number of winged male and female deer keds were found positive for these pathogens. Therefore, *L. cervi* is probably not a vector for *A. phagocytophilum*, and *Rickettsia* species.

*Bartonella schoenbuchensis* is vertically transmitted from wingless females to developing larvae. It is very likely that *L. cervi* is a vector for *Bartonella,* including the zoonotic pathogen *B. schoenbuchensis,* because we identified this zoonotic pathogen in dissected developing larvae and free ranging winged *L. cervi* males and females.
